# FHL1 Inhibits the Progression of Colorectal Cancer by Regulating the Wnt/β-Catenin Signaling Pathway

**DOI:** 10.7150/jca.60543

**Published:** 2021-07-03

**Authors:** Yujing Liu, Chunyan Wang, Peiqiu Cheng, Shengan Zhang, Wenjun Zhou, Yangxian Xu, Hanchen Xu, Guang Ji

**Affiliations:** 1 Institute of Digestive Diseases, Longhua Hospital, Shanghai University of Traditional Chinese Medicine, Shanghai 200032, China.; 2 Shanghai University of Traditional Chinese Medicine, Shanghai 200032, China.; 3 Department of General Surgery, Longhua Hospital, Shanghai University of Traditional Chinese Medicine, Shanghai 200032, China.

**Keywords:** Colorectal cancer, FHL1, RNA sequencing, Wnt/β-catenin signaling pathway

## Abstract

**Purpose:** This study aims to explore the FHL1 expression level in colorectal cancer (CRC) patients, analyze its association with patient survival and investigate the role of FHL1 in CRC.

**Methods:** We used secondary sequencing to profile mRNA expression in CRC tissue and corresponding adjacent normal tissue from four CRC patients. We focus on FHL1 and analyzed the association between its expression level and clinical indicators. Furthermore, we explored the functional role of FHL1 in colorectal cancer tumorigenesis by transfecting cells with siRNA or overexpression plasmids.

**Results:** Hierarchical clustering revealed significantly differentially expressed mRNAs. FHL1 expression was significantly lower in CRC tissue than in adjacent normal tissue as well as in CRC cell lines relative to NCM460. Low FHL1 expression in CRC tissue correlated with poor patient survival. Our data demonstrated that overexpression of FHL1 inhibited the proliferation, colony formation potential, and expression of CdK4 and Cyclin D1, whereas ablating FHL1 promoted their proliferation and colony formation potential, suggesting that FHL1 acts as a tumor suppressor in CRC. Moreover, we showed that FHL1 inhibited the proliferation of colorectal cancer cells by negatively regulating the Wnt/β-catenin signaling pathway.

**Conclusion:** FHL1 is a potential tumor suppressor gene in colorectal cancer, and regulation of the FHL1-Wnt/β-catenin pathway may be part of its antitumor mechanism.

## Introduction

Colorectal cancer (CRC) is the third most common cancer in the world, causing approximately 600,000 deaths worldwide every year 1. Although great progress has been made in the diagnosis and treatment of CRC, the majority of CRC patients are diagnosed at an advanced stage and have a poor prognosis. The 5-year survival rate of patients with advanced colorectal cancer is only approximately 10% 2. Therefore, it is important to elucidate the molecular mechanisms of colorectal cancer. Continuous exploration of the pathogenesis of this cancer can provide a better strategy for treating CRC.

The Wnt signaling pathway is a key mediator of tissue homeostasis and repair and is frequently mutated during tumor development. More than 90% of colorectal cancer patients demonstrate hyperactivation of the Wnt pathway, which is considered to be an initiating and driving event in tumor development 3. The Wnt/β-catenin signaling pathway is a classic Wnt signaling pathway. High activation of the Wnt/β-catenin signaling pathway promotes cell growth and is an important factor in the development of various tumors 4. Excessive activation of the Wnt/β-catenin signaling pathway leads to intracellular accumulation of β-catenin, which forms a complex with intracellular TCF/LEF transcription factors, promoting the expression of downstream target genes. Thus, changes in biological functions, such as cell proliferation, will eventually lead to the occurrence of tumors 5,6.

FHL1 belongs to the FHL (four and a half LIM domains) family, which consists primarily of FHL1, FHL2, FHL3, FHL4 and FHL5/ACT. The family is characterized by four and a half N-terminal LIM domains that regulate proliferation, differentiation, apoptosis, adhesion, migration, transcription and other cellular processes 7. Studies have shown that FHL1 is downregulated in a variety of cancers, such as gastric cancer, lung cancer and oral cancer 8-10. Regarding the regulatory relationship between FHL1 and the Wnt signaling pathway, studies in mouse C2C12 muscle cells demonstrated that the β-catenin protein could significantly activate the FHL1 promoter, thus inducing muscle cell differentiation 11. Another study suggested that FHL1 may act as a link between estrogen and the Wnt signaling pathway in osteoblasts 12. However, the relationship between FHL1 and the Wnt signaling pathway in CRC remains unclear.

In this study, we explored the functional role of FHL1 in CRC, elucidated its expression and determined the associated with the Wnt/β-catenin signaling pathway in CRC cells.

## Materials and methods

### Patients and tissue samples

We collected 40 pairs of primary colorectal cancer tissue and adjacent normal tissue from patients undergoing surgery at Longhua Hospital Affiliated to Shanghai University of Traditional Chinese Medicine. No patients received radiotherapy or chemotherapy before surgery. All fresh specimens were immediately stored at -80 °C in an ultra-low-temperature freezer for preservation. At the same time, clinical information and medical records for the patients were collected, including name, sex, and age, as well as primary part, size, histological type, degree of differentiation, and clinical pathological grade of the tumor. Collection of tissue specimens was approved by the ethics committee of Shanghai University of Traditional Chinese Medicine. The tumor tissue microarray, which contained 177 paired CRC and adjacent normal tissues, was purchased from Shanghai Outdo Biotech Co., Ltd**.**

### RNA Extraction and RNA Sequencing

Total RNA was extracted from five colorectal adenoma, five CRC, and five adjacent normal tissues using TRIzol reagent (Life Technologies CA, USA) following the manufacturer's instructions. The RNA quality was assessed by spectrophotometry and denaturing agarose gel electrophoresis. The sequencing libraries for mRNAs were prepared, purified, qualified and sequenced using Illumina Hiseq 4000. Clean reads were obtained and aligned to the human genome. Significantly differentially expressed RNAs were identified using DEseq2 software with a threshold of 2-fold change and p value less than 0.05.

### Cell lines, cell culture and siRNA transfection

Human colorectal cancer cell lines, namely, HCT116, HT29, LOVO, SW620 and SW480, were purchased from the Cell Bank of the Chinese Academy of Sciences (Shanghai, China). A normal colon epithelial cell line, NCM460, was kindly provided by the Institute of Chinese Medicine of Shanghai University of Traditional Chinese Medicine. These colorectal cancer cell lines, except for HT29, were maintained in RPMI 1640 medium supplemented with 10% fetal bovine serum (FBS), 100 units/ml penicillin, and 100 mg/ml streptomycin. HT29 and NCM460 cells were cultured in McCoy′s 5A medium supplemented with the same additions. The siRNA molecules and overexpression lentiviral vector were designed by Shanghai Genechem Co., Ltd. To construct the FHL1 recombinant adenovirus vector, the full-length FHL1 ORF was inserted into the stratagene plasmid (pShuttle-IRES-hrGFP-1). The novel recombinant plasmid, Ad-GFP-FHL1, was verified by restriction endonuclease digestion and sequencing. Transfections into 293T cells for lentivirus packaging were performed using Lipofectamine 2000 (Invitrogen) according to the manufacturer's recommendations into 293T cells for lentivirus packaging. CRC cells were harvested 48 hours after transfection with lentivirus and were selected with 1 μg/ml puromycin to obtain a stable cell line.

### Cell growth and cell viability assay

Stably transfected cells were seeded at different densities (HCT116, 5 × 10^3^; HT29, 1.5 × 10^4^) onto 96-well plates in 100 µl of medium. At 24 h, 48 h and 72 h, cell viability was measured using a Cell Counting Kit-8 (Dojindo Laboratories, Kumamoto, Japan) according to manufacturer's instructions. The experiment was performed using eight replicates.

### RNA extraction and qRT-PCR

Total RNA was extracted using TRIzol reagent. Two microliters of total RNA was used for reverse transcription (RT) with an ABI Reserve Transcription Kit (ABI, USA). To quantify the gene expression levels of FHL1, real-time quantitative PCR was conducted on a StepOne Plus Real-Time PCR System (ABI, USA) using SYBR® Green PCR Master Mix (Applied Biosystems) according to the manufacturer's instructions. Primer sequences were as follows: FHL1 (forward) 5′-CTGGGTTTGGTAAAGGCTCC-3′; FHL1 (reverse) 3′-GGCACAGTCGGGACAATACAC-5′; β-actin (forward) 5′-GAGACCTTCAACACCCCAGC-3′; β-actin (reverse) 3′-ATGTCACGCACGATTTCCC-5′; LEF1 (forward) 5′- GGATCACACCCGTCACACATC -3′; LEF1 (reverse) 3′- TGGGTAGGGTTGCCTGAATC -5′; TCF4 (forward) 5′- GTCTGGGCTCAGGGTATGG -3′; and TCF4 (reverse) 3′- CAGGTCAGGGGAAGTCGC -5′.

### Protein extraction and Western blot

Protein extraction was performed using RIPA lysate (Beyotime Institute of Biotechnology). Anti-FHL1 rabbit antibody (Ab133661, Abcam, 1:1000), anti-CDK4 rabbit antibody (ET1612-1, Huabio, 1:1000), anti-Cyclin D1 rabbit antibody (2922S, Cell Signaling Technology, 1:1000), anti-active-β-catenin rabbit antibody (19807, Cell Signaling Technology, 1:1000), anti-GSK rabbit antibody (12456S, Cell Signaling Technology, 1:1000), anti-GSK-3β rabbit antibody (9323S, Cell Signaling Technology, 1:1000), anti-LEF1 rabbit antibody (14972-1-AP, Proteintech, 1:1000), anti-TCF4 rabbit antibody (22337-1-AP, Proteintech, 1:1000), and anti-β-actin rabbit antibody (R1207-1, Huabio, 1:2000) were used as primary antibodies for the detection of specific proteins.

### Immunohistochemistry

Immunohistochemical staining of tissue was performed using the polymer HRP detection system on formalin-fixed, paraffin-embedded tissue sections. The paraffin sections were dewaxed, subjected to antigen retrieval, blocked with endogenous peroxidase, incubated with goat serum, incubated with primary antibodies, incubated with HRP-conjugated secondary antibodies, stained with DAB, counterstained with hematoxylin, and dehydrated in sequence. The dilution of primary antibody against FHL1 (Ab133661, Abcam) was 1:200. The immunoreactivity score was defined as the product of the extent score and the intensity score. A score of ≤3 was defined as negative, and a score of >3 was defined as positive.

### Statistical analysis

All experimental data are expressed as means ± SDs and were analyzed using SPSS 21.0 software and plotted by GraphPad Prism 6.0 software. After a test for variance homogeneity, an independent-samples t test was used to assess significant differences between groups, and one-way ANOVA was used for multiple groups. The Kaplan-Meier method was used for survival analysis. A p-value of less than 0.05 was considered statistically significant.

## Results

### FHL1 expression was downregulated in colorectal cancer tissues and associated with a worse clinical outcome

**Figure 1:**
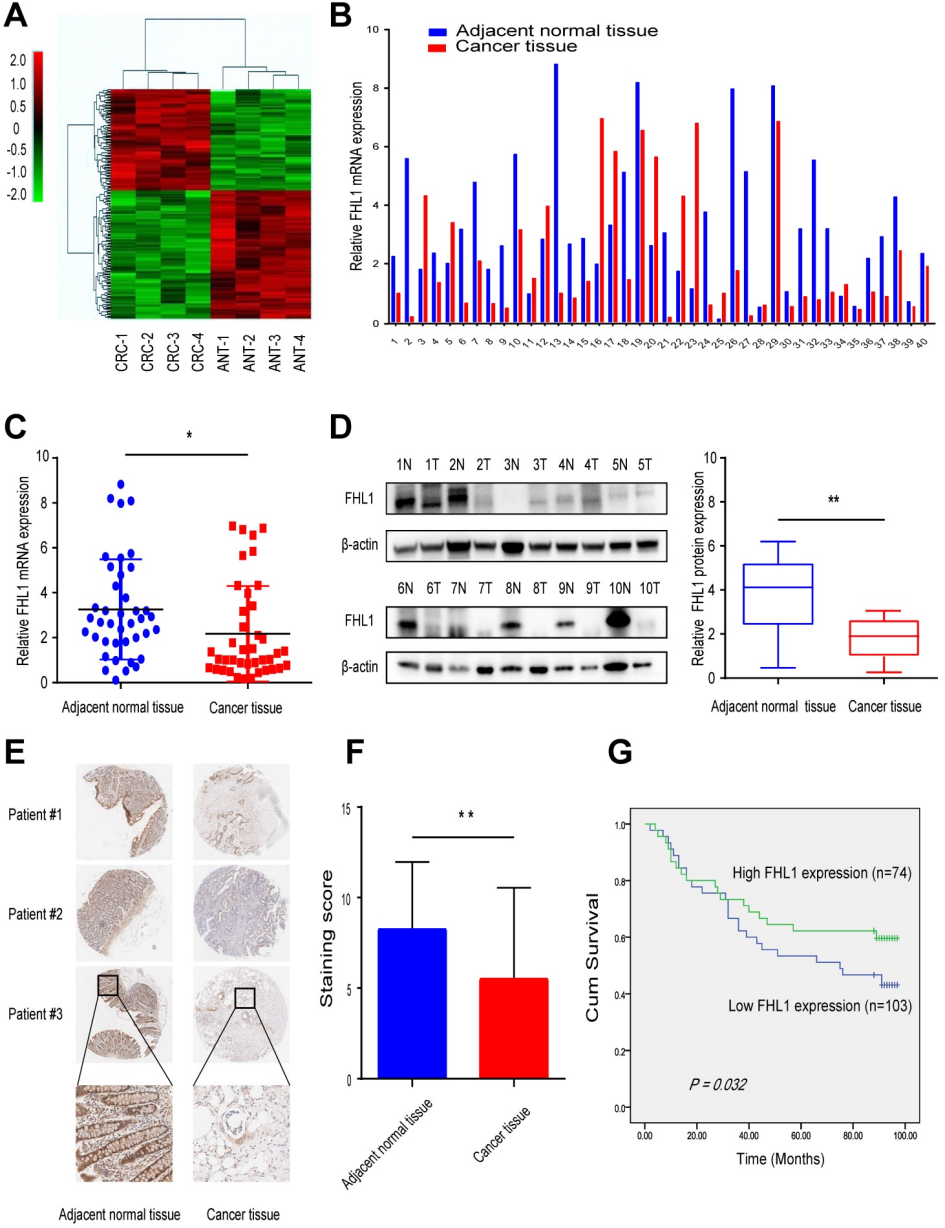
** Downregulation of FHL1 was identified in colorectal cancer tissues. A.** RNA expression profiles in 4 paired CRC tissues and the corresponding adjacent normal tissues. **B,C.** FHL1 mRNA expression levels in 40 paired CRC tissues and the corresponding adjacent normal tissues were measured by using qRT-PCR. **D.** FHL1 protein levels in 8 representative CRC tissues and the corresponding adjacent normal tissues were tested by Western blot. **E.** Representative IHC staining images of FHL1 in a tissue microarray that contained 177 paired CRC and adjacent normal tissues (scale bar, 100 µm). **F.** Comparison of the relative FHL1 expression levels in CRC tumors compared to adjacent normal tissues in the tissue microarray. **G.** Kaplan-Meier survival curve indicating overall survival in relation to FHL1 expression levels in CRC tumor tissues from the tissue microarray. Data represent the mean ± SD. * *P* < 0.05, ** *P* < 0.01.

First, we used high-throughput RNA sequencing to profile mRNA expression in the CRC tissue and corresponding adjacent normal tissue from four CRC patients. Hierarchical clustering revealed significantly differentially expressed mRNAs (Fig. 1A). By analyzing the sequencing results, we found that FHL1 was one of the genes with a significant difference in expression level between the two groups (Table 1). These results showed that FHL1 expression in colon-adjacent normal tissues was significantly higher than that in CRC tissues. Next, FHL1 mRNA expression was detected in 40 CRC tissue samples and paired adjacent normal tissue. The results of qRT-PCR analysis were consistent with the previous results that FHL1 mRNA expression was higher in colon-adjacent normal tissue than in CRC tissue (*P* < 0.05, Fig. 1B and C). We also explored the associations between FHL1 mRNA expression level and the clinicopathological features of these 40 colorectal cancer patients. As shown in Table 2, FHL1 expression did not significantly differ with age, gender, tumor size, tumor location or distant metastasis (all *P* > 0.05). However, FHL1 expression was negatively correlated with advanced pT stage (*P* = 0.003) and lymph node metastasis (*P* = 0.004), indicating that low FHL1 expression was correlated with adverse clinicopathological features. Meanwhile, aberrant low expression of FHL1 in CRC tissues was also confirmed by Western blot using adjacent normal and tumor tissue samples (*P* < 0.05, Fig. 1D). Furthermore, we performed immunohistochemical (IHC) analysis using a tumor tissue microarray that included 177 paired CRC and adjacent normal tissues (Fig. 1E). The expression of FHL1 was scored according to staining intensity and the proportion of signals in each sample. In comparison with CRC tissues, the adjacent normal tissues had higher expression levels of FHL1 (*P* < 0.01, Fig. 1F). Based on the IHC results from the tissue microarray, we performed survival analysis. As shown in Fig. 1G, low expression of FHL1 was significantly associated with poor survival of patients with CRC. All these results indicated that FHL1 expression was downregulated in colorectal cancer tissues and that this was associated with a worse clinical outcome.

### FHL1 inhibited colon cancer cell proliferation *in vitro*

**Figure 2:**
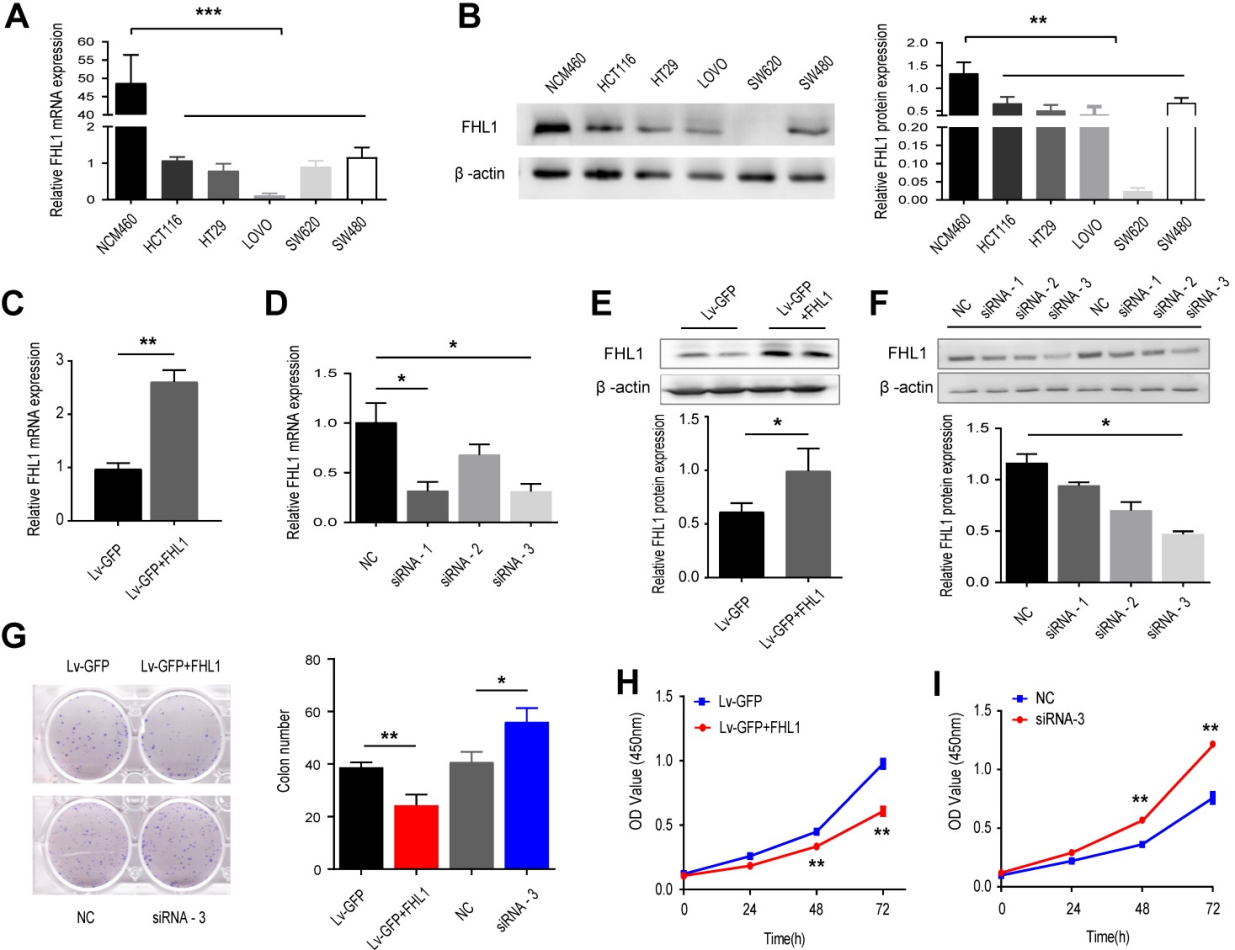
** Effects of FHL1 overexpression or knockdown on aggressive proliferation of colorectal cancer cells were studied. A,B.** FHL1 mRNA and protein levels were measured in 5 CRC cell lines and normal colon epithelial cells by qRT-PCR (**A**) and Western blot (**B**). **C, E.** FHL1 gene expression and protein levels were evaluated in HT-29 cells after transfection with the overexpression plasmid. **D, F.** The silencing effect on FHL1 by three siRNAs was evaluated in HCT-116 cells using qRT-PCR (**D**) and Western blot (**F**). **G.** The colony formation potential of HCT-116 and HT-29 cells after FHL1 knockdown by siRNA-3 or FHL1 overexpression by Lv-GFP+FHL1 was analyzed with a colony formation assay. **H-I.** The proliferation of HCT-116 and HT-29 cells after FHL1 knockdown by siRNA-3 or FHL1 overexpression via Lv-GFP+FHL1 was measured using a CCK-8 cell-counting kit. Data are presented as mean ± SD and are representative of 3 independent experiments. * *P* < 0.05, ** *P* < 0.01, *** *P* < 0.001.

Before we explored the functional role of FHL1 in colorectal cancer tumorigenesis, we measured the expression levels of FHL1 mRNA and protein in 5 CRC cell lines, namely, HCT116, HT29, LOVO, SW620 and SW480, as well as in NCM460, a normal colon epithelial cell line. The results showed that both FHL1 mRNA and protein expression levels were downregulated in the five colorectal cancer cell lines compared with NCM460 (Fig. 2A and B). We chose HCT116 and HT29 cells for further study and transfected them with either siRNA or an overexpression plasmid in order to knock down or overexpress FHL1 in HCT116 and HT29 cells, respectively. After lentivirus infection, the knockdown and overexpression efficiencies of FHL1 were confirmed by qRT-PCR and Western blot. Of the three siRNAs used, siRNA-3 had the best silencing effect and significantly decreased FHL1 mRNA and protein levels in HCT116 cells (Fig. 2D and F). The lentiviral vector-GFP containing FHL1 (Lv-GFP-FHL1) successfully increased FHL1 expression in HT29 cells (Fig. 2C and E). The results from the CCK-8 proliferation assay and colony formation assay showed that silencing FHL1 significantly promoted cell growth (Fig. 2G) and increased the number of colonies present (Fig. 2I). Conversely, FHL1 overexpression in HT29 cells had the opposite effect (Fig. 2H and J). These results indicated that knockdown of FHL1 expression promoted the proliferation of colorectal cancer cells, thereby potentially promoting tumor oncogenesis and progression.

### FHL1 negatively regulates the Wnt/β-catenin pathway

**Figure 3:**
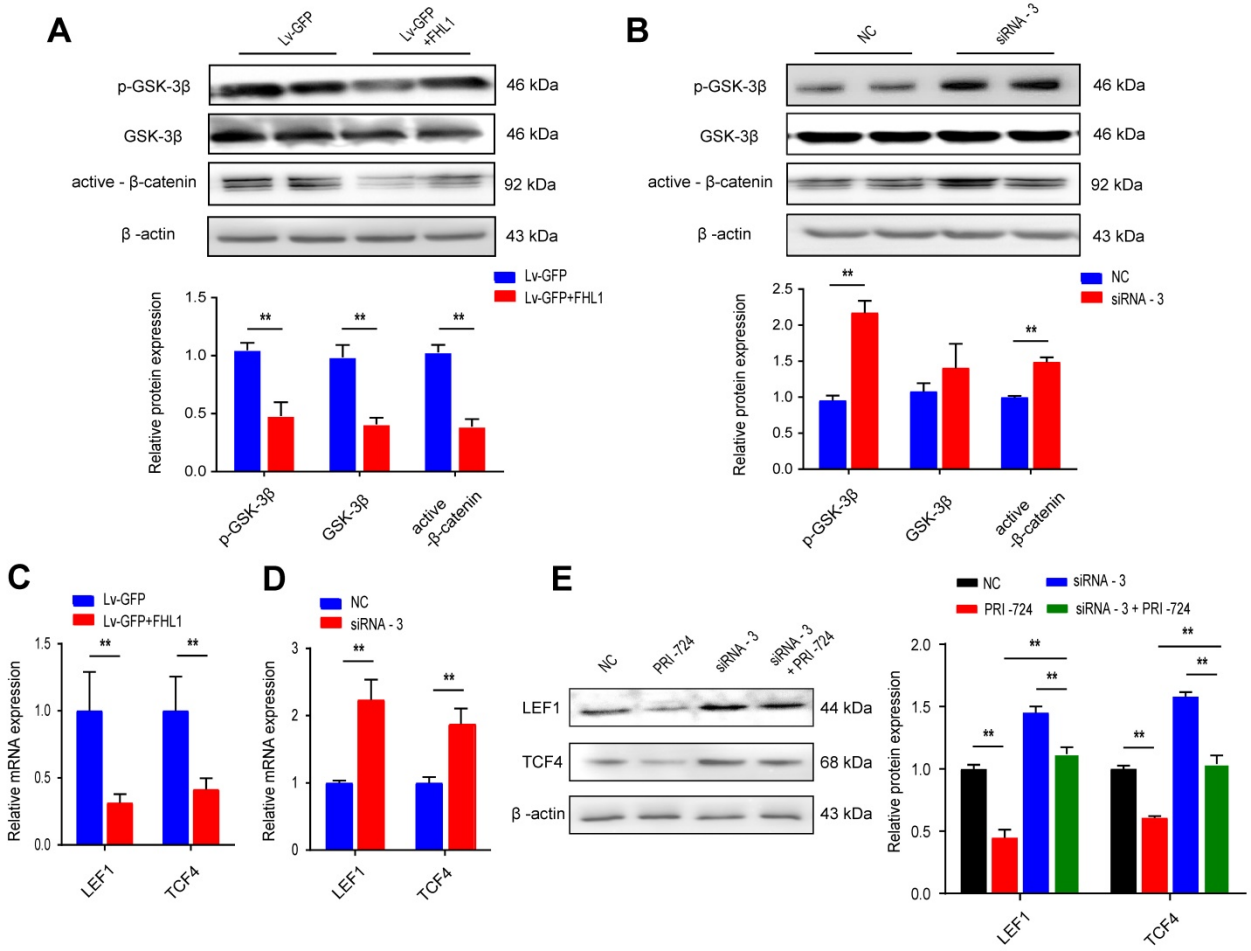
** FHL1 negatively regulates the Wnt/β-catenin pathway. A-B.** The p-GSK, GSK and β-catenin protein levels were measured by Western blot after FHL1 knockdown with siRNA-3 or FHL1 overexpression with Lv-GFP+FHL1 in HCT-116 cells and HT-29 cells, respectively. **C-D.** Real-time PCR analysis of the expression levels of LEF1 and TCF4 in FHL1 knockdown or overexpression cells. **E.** The LEF1 and TCF4 protein levels were assessed by Western blot after using FHL1 siRNA-3 or/and PRI-724 in HCT-116 cells. Data are presented as mean ± SD and are representative of 3 independent experiments. * *P* < 0.05, ** *P* < 0.01.

**Figure 4:**
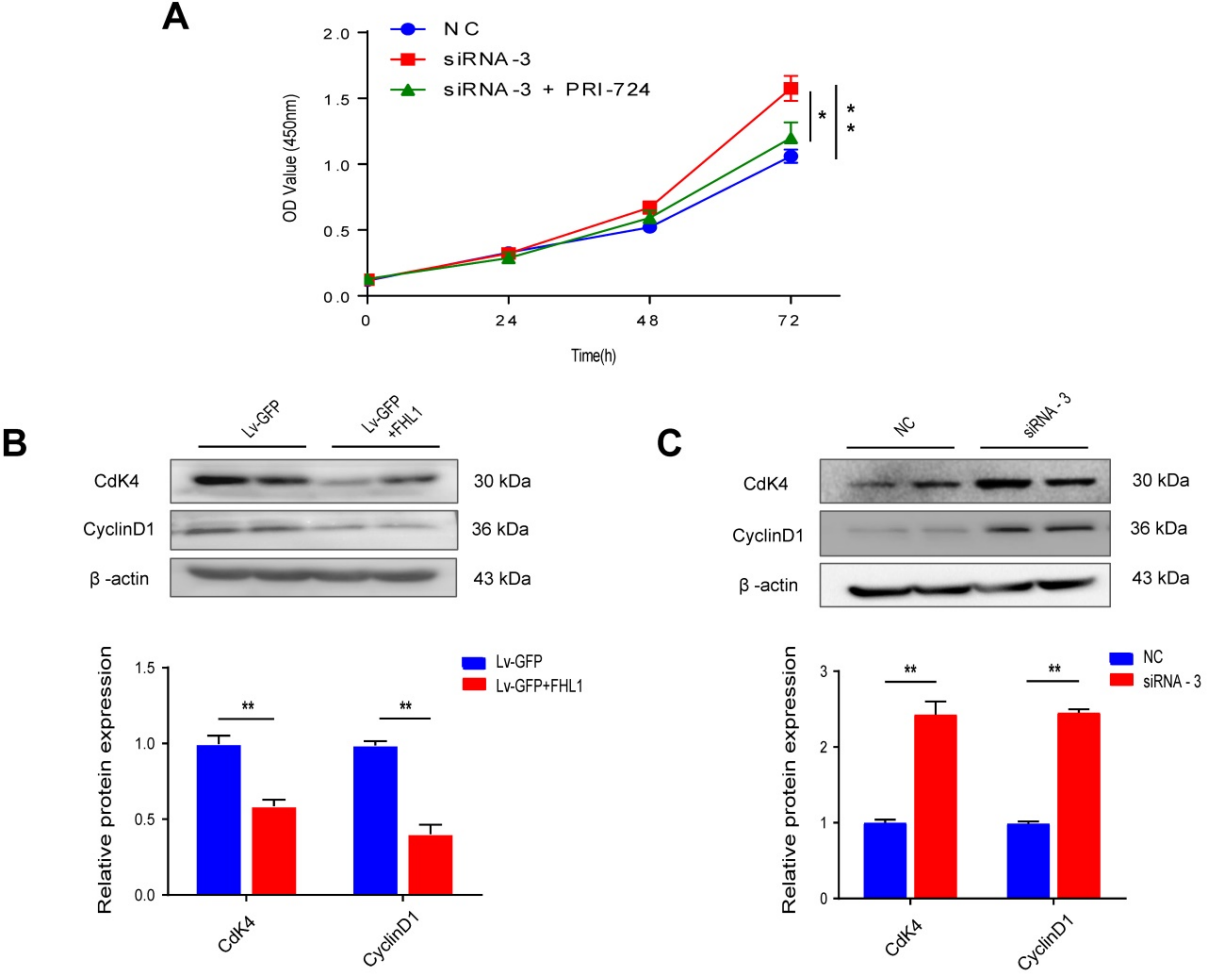
** The effect of FHL1 overexpression or knockdown on cell cycle proteins in colorectal cancer cells was elucidated. A.** The proliferation of HCT-116 cells after FHL1 knockdown by siRNA-3 or treatment with PRI-724 was measured using a CCK-8 cell-counting kit. **B-C.** Expression of cell cycle-related proteins was measured by Western blot after FHL1 knockdown with siRNA-3 or FHL1 overexpression with Lv-GFP+FHL1 in HCT-116 cells and HT-29 cells, respectively. Data are presented as mean ± SD and are representative of 3 independent experiments. * *P* < 0.05, ** *P* < 0.01.

Canonical Wnt signaling plays a crucial role in the progression of colorectal cancer by regulating proliferation, differentiation, and cell fate decisions. Previous studies have shown the regulatory relationships between FHL1 and the Wnt pathway in myocyte differentiation and bone formation. We further evaluated this relationship by overexpressing or knocking down FHL1 in colon cancer cells. HT29 cells with FHL1 overexpression showed marked decreases in GSK3β phosphorylation and active β-catenin (Fig. 3A), indicating inactivation of the Wnt pathway. The mRNA expression levels of molecules downstream of the Wnt/β-catenin pathway (LEF1 and TCF4) were also measured. FHL1 overexpression in HT29 cells notably inhibited the expression of LEF1 and TCF4 mRNA (Fig. 3C). As shown in Fig. 3, silencing of FHL1 in HCT116 cells showed the opposite effects on the Wnt/β-catenin pathway (Fig. 3B, D). Additionally, PRI-724, an inhibitor of the Wnt/β-catenin pathway, was used to verify the relationship between FHL1 and this pathway. As shown in Fig. 3E, inhibition of the Wnt/β-catenin pathway partly reversed the upregulation of LEF1 and TCF4 protein levels after FHL1 siRNA treatment in HCT-116 cells. The results of the CCK-8 assay indicated that the promotion of proliferation by FHL1 knockdown could be rescued by the inhibitor PRI-724 in HCT116 cells (Fig. 4A). These results revealed that FHL1 can negatively regulate the Wnt/β-catenin pathway.

### FHL1 regulates the expression of CDK4 and Cyclin D1

**Figure 5:**
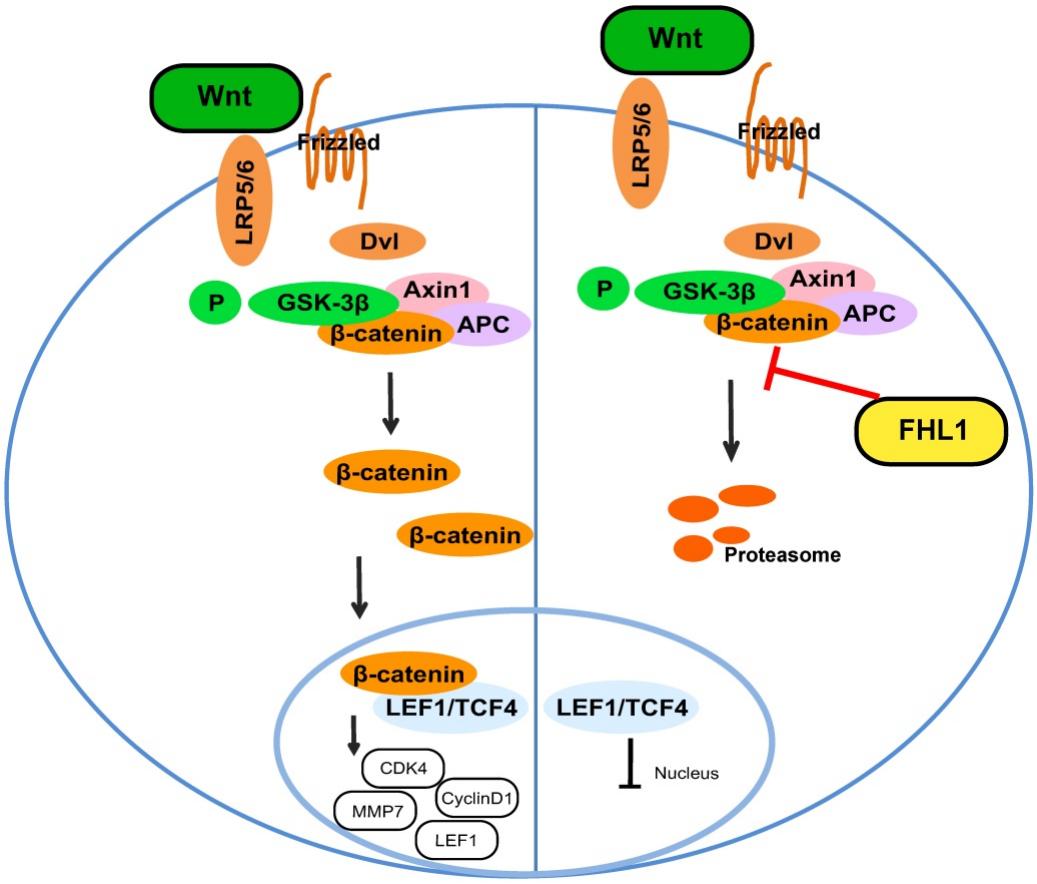
Schematic illustration of FHL1 inhibition of the Wnt/β-catenin pathway in colorectal cancer cells.

Our results proved that FHL1 can negatively regulate the Wnt/β-catenin pathway. Previous studies have shown that the Wnt/β-catenin pathway can affect tumor cell growth by regulating the expression of cyclin and CDK, so we measured these cell cycle proteins. As shown in Fig. 4B and C, FHL1 overexpression in HT29 cells inhibited the expression of cyclin D1 and CDK4 (Fig. 4B), whereas silencing of FHL1 in HCT116 cells greatly increased the expression of cyclin D1 and CDK4 (Fig. 4C). These results suggest that FHL1 may affect colorectal cancer cell growth by regulating cell cycle proteins. Taken together, these data indicate that FHL1 inhibits the proliferation of colorectal cancer cells by regulating the Wnt/β-catenin signaling pathway (Fig. 5).

## Discussion

Colorectal cancer is one of the most common malignant tumors, and it is important to screen for effective new CRC biomarkers and to explore CRC pathogenesis-related signaling pathways. As a member of the FHL family, FHL1 is characterized by four and a half LIM domains at the N-terminus. The LIM domain is a zinc finger structure rich in cysteine that could be involved in various protein-protein interactions. By interacting with other proteins, FHL family proteins can regulate cell proliferation, differentiation, apoptosis, adhesion, migration, transcription and signal transduction 13-16. Previous studies on FHL1 have shown that its mutation leads to abnormal muscle development, and the expression of FHL1 is upregulated in pathological myocardial hypertrophy and cardiomyopathy 17-19. Regarding FHL1 expression in tumors, studies have shown that FHL1 is downregulated in various tumors, such as breast, lung, oral and bladder cancer 20-22. Our study evaluated the expression of FHL1 in colorectal cancer. Compared with that in adjacent tissues, the expression of FHL1 mRNA and protein in colorectal cancer tissue was significantly downregulated, and it was correlated with TNM stage and lymph node metastasis in CRC patients. Meanwhile, immunohistochemical staining showed that patients providing tissue samples with low expression of FHL1 had poor survival. In order to validate our data indicating the low expression of FHL1 in CRC tumor tissue, we also checked the correlation between FHL1 expression and survival in TCGA database. FHL1 gene expression in colorectal cancer tissues was also significantly downregulated (Fig. S1). These data suggest that FHL1 has a tumor-suppressive effect in CRC.

Next, we selected 5 colorectal cancer cell lines and 1 normal intestinal epithelial cell line and detected the gene and protein expression levels of FHL1 in each. Compared with those in the normal intestinal epithelial cell line, NCM460, the gene and protein expression levels of FHL1 in all 5 CRC cell lines were significantly reduced. Functional experiments further confirmed that FHL1 inhibited the proliferation of CRC cells *in vitro*. Our data demonstrated that overexpression of FHL1 inhibited the proliferation, colony formation potential, and expression of CdK4 and Cyclin D1, whereas ablating FHL1 promoted proliferation and colony formation potential, suggesting that FHL1 acts as a tumor suppressor in CRC. In normal human cells, cell division is an orderly, tightly regulated process that includes multiple checkpoints in the cell cycle to ensure genomic integrity. Cyclins and their associated CDKs are central proteins that regulate cell cycle progression 22. These results are consistent with those of previous studies showing that FHL1 inhibits the proliferation of lung cancer cells *in vitro* 9. Studies in oral cancer and bladder cancer have also found that reduced expression of FHL1 promotes tumor progression and is associated with poor prognosis 10,23. These studies strongly suggest that FHL1 plays an anticancer role in the proliferation of a variety of tumor types.

Overactivation of Wnt signaling is considered one of the most important mechanisms in tumorigenesis and development 24. There is considerable evidence that activated Wnt signaling is associated with tumorigenesis in various human cancers 25-27. Another protein in the FHL family, FHL2, has been shown to regulate transcription of the Wnt pathway by interacting with β-catenin and by activating T cytokine-dependent transcription and expression of Cyclin D1 and IL-8 promoters in renal and colon cell lines 28,29. Previous studies of the relationship between FHL1 and Wnt signaling showed that Wnt signaling could control bone formation by regulating the activity of FHL1, and FHL1 may be a key molecule that mediates the relationship between Wnt signaling and estrogen signaling during bone formation 12. The regulatory relationship between the FHL family and the Wnt pathway is not consistent among disease studies. The Wnt/β-catenin signaling pathway is a classic Wnt signaling pathway. Currently, degeneration of the Wnt/β-catenin signaling pathway is known to exist in approximately 90% of colon cancer patients, which is of great significance in the initiation and development of colorectal cancer. In this study, we revealed the relationship between FHL1 and the Wnt/β-catenin signaling pathway by *in vitro* experiments on colorectal cancer cells. The results showed that overexpression of FHL1 could significantly inhibit the expression of p-GSK-3, active-β-catenin and the downstream molecules LEF1 and TCF4 in the Wnt/β-catenin signaling pathway in CRC cells. These results suggest that FHL1 can regulate the activation of the Wnt/β-catenin signaling pathway. Lymphoid enhancer factor/T cell factor proteins (LEFs/TCFs) mediate Wnt signals in the nucleus by recruiting β-catenin and its coactivators to Wnt response elements (WREs) of target genes. Misregulation of β-catenin plays a role in diseases such as cancer, where overactive Wnt signaling drives LEFs/TCFs to transform cells 30. By using the Wnt signaling pathway inhibitor PRI-724 to inhibit the activation of the Wnt/β-catenin signaling pathway, we found that PRI-724 could partially reverse the activation of FHL1 on Wnt/β-catenin and reduce the expression of the downstream molecules LEF1 and TCF4, indicating that the Wnt/β-catenin signaling pathway is one of the important pathways by which FHL1 exerts its anticancer effect.

## Conclusions

In summary, our study demonstrates that FHL1 exhibits an anticancer function and that its inhibition of Wnt/β-catenin signaling is one of the mechanisms by which FHL1 plays its role. FHL1 expression is an independent predictor of the prognosis of patients with CRC.

## Supplementary Material

Supplementary figure S1.

## Figures and Tables

**Table 1:** The top 10 upregulated and downregulated mRNAs from the RNA-seq

Gene name		Fold changes	*P*-value
Endothelial Cell-specific Molecular 1	ESM1	12.31	0.0089
Forkhead Box Q1	FOXQ1	11.72	0.0171
Tropomyosin 4	TPM4	9.59	0.0137
Tescalcin	Tescalcin	9.21	0.0392
PR Domain Zinc Finger Protein 13	PRD13	6.65	0.0023
Matrix Metallopeptidase 28	MMP28	6.52	0.0243
Adenylate Cyclase 9	ADCY9	5.54	0.0217
Purinergic Receptor P2Y13	P2RY13	5.47	0.0291
ER Membrane Protein Complex Subunit 1	EMC1	5.11	0.0337
Frizzled Class Reptor 3	FZD3	5.03	0.0415
Transmembrane and immunoglobulin domain-containing protein 1	TMIGD1	-12.35	0.0423
Guanylate cyclase activator 2B	GUCA2B	-11.48	0.0216
Four and a Half LIM Domains 1	FHL1	-11.34	0.0062
Carbonic anhydrase 1	CA1	-8.43	0.0415
Bone morphogenetic protein 3	BMP3	-6.94	0.0243
Ras-related and estrogen-regulated growth inhibitor-like protein	RERGL	-6.78	0.0221
XK-related protein 4	XKR4	-5.91	0.0131
Proline-rich membrane anchor 1	PRIMA1	-5.67	0.0079
Kv channel-interacting protein 4	KCNIP4	-5.41	0.0235
Peptidase inhibitor 16	PI16	-5.31	0.0212

**Table 2:** Associations between FHL1 mRNA levels and clinical parameters in a training cohort (n = 40)

Characteristic	No. of patients (%)	FHL1 mRNA expression	*P* value (2^-ΔCt^ Mean ± SD)
**Age, y**	40 (100)		
≥60	31 (77.5)	2.440±2.266	0.137
< 60	9 (22.5)	1.237±1.214	
**Sex**	40 (100)		
Men	27 (67.5)	2.039±2.004	0.583
Women	13 (32.5)	2.440±2.417	
**Tumor size**	40 (100)		
≥5 cm	18 (45)	2.190±2.257	0.957
< 5 cm	22 (55)	2.153±2.047	
**Tumour location**	40 (100)		
Left hemicolon	34 (85)	2.178±2.108	0.951
Right hemicolon	6 (15)	2.120±2.418	
**pT stage**	40 (100)		
I-II	20 (50)	3.118±2.537	**0.003**
III-IV	20 (50)	1.221±0.964	
**Lymph node metastasis**	40 (100)		
pN positive	19 (47.5)	1.194±0.851	**0.004**
pN negative	21 (52.5)	3.053±2.534	
**Distant metastasis**	40 (100)		
pM positive	6 (15)	0.902±0.840	0.114
pM negative	34 (85)	2.393±2.209	

## Abbreviations

CRC: colorectal cancer
FHL-1: four and a half LIM domains 1
LEF1: lymphoid enhancer binding factor 1
TCF4: transcription factor 4
CDK4: cyclin dependent kinase 4
