# Supplementary figures and images for: FHL1 Inhibits the Progression of Colorectal Cancer by Regulating the Wnt/β-Catenin Signaling Pathway
Source: J Cancer. 2021 Jul 3;12(17):5345–54. doi: 10.7150/jca.60543 (PMC8317513; doi:10.7150/jca.60543)

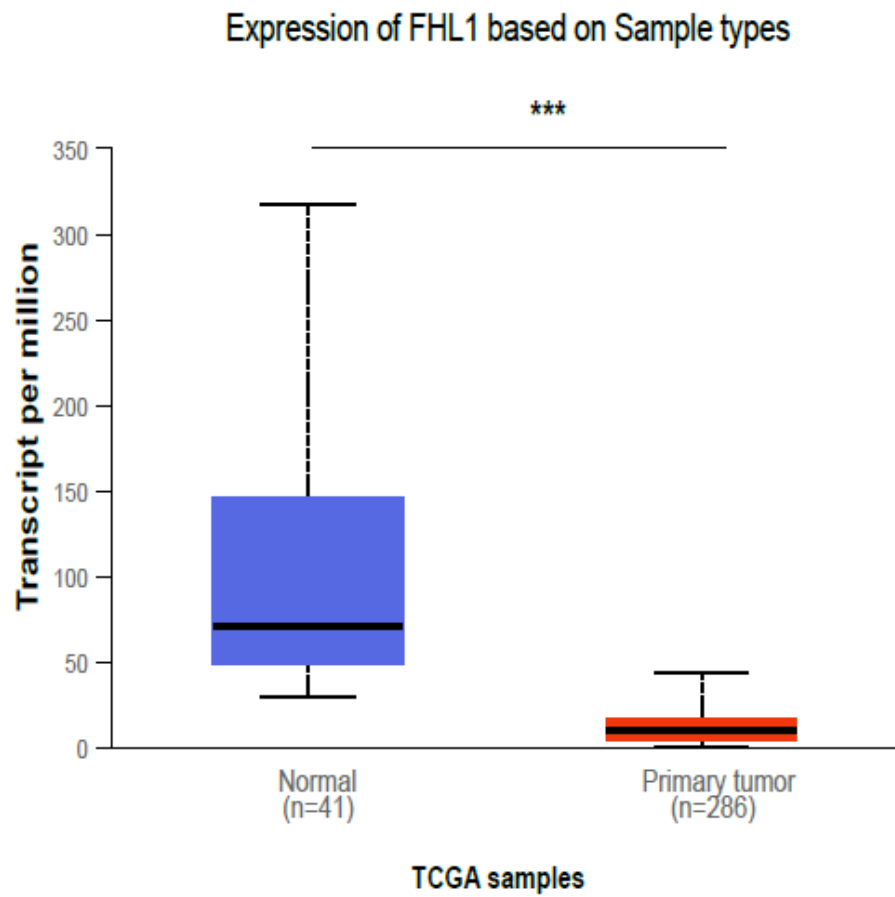

Fig.S1 FHL1 expression in colorectal cancer tissues and normal tissues from TCGA database.

Supplement: Supplementary file 1 — Supplementary figure S1. [file jcav12p5345s1.pdf]
